# DNA methylation of oestrogen-regulated enhancers defines endocrine sensitivity in breast cancer

**DOI:** 10.1038/ncomms8758

**Published:** 2015-07-14

**Authors:** Andrew Stone, Elena Zotenko, Warwick J. Locke, Darren Korbie, Ewan K. A. Millar, Ruth Pidsley, Clare Stirzaker, Peter Graham, Matt Trau, Elizabeth A. Musgrove, Robert I. Nicholson, Julia M. W. Gee, Susan J. Clark

**Affiliations:** 1 Epigenetics Research Program, Genomics and Epigenetics Division, Garvan Institute of Medical Research, Sydney, New South Wales 2010, Australia; 2 Faculty of Medicine, St Vincent's Clinical School, UNSW, NSW 2052 & St Vincent's Hospital, Sydney, New South Wales 2010, Australia; 3 Australian Institute for Bioengineering and Nanotechnology, University of Queensland, Brisbane, Queensland 4072, Australia; 4 Translational Breast Cancer Research, The Kinghorn Cancer Centre, Garvan Institute of Medical Research, Sydney, New South Wales 2010, Australia; 5 Department of Anatomical Pathology, South Eastern Area Laboratory Service, St George Hospital, Kogarah, Sydney, New South Wales 2217, Australia; 6 School of Medicine and Health Sciences, University of Western Sydney, Campbelltown, Sydney, New South Wales 2560, Australia; 7 Faculty of Medicine, UNSW, Kensington, New South Wales 2052, Australia; 8 Department of Radiation Oncology, Cancer Care Centre, St George Hospital, Kogarah, Sydney, New South Wales 2217, Australia; 9 Wolfson Wohl Cancer Research Centre, University of Glasgow, Glasgow G61 1BD, UK; 10 Breast Cancer Molecular Pharmacology Group, School of Pharmacy and Pharmaceutical Sciences, Cardiff University, Wales CF10 3NB, UK

## Abstract

Expression of oestrogen receptor (ESR1) determines whether a breast cancer patient receives endocrine therapy, but does not guarantee patient response. The molecular factors that define endocrine response in ESR1-positive breast cancer patients remain poorly understood. Here we characterize the DNA methylome of endocrine sensitivity and demonstrate the potential impact of differential DNA methylation on endocrine response in breast cancer. We show that DNA hypermethylation occurs predominantly at oestrogen-responsive enhancers and is associated with reduced ESR1 binding and decreased gene expression of key regulators of ESR1 activity, thus providing a novel mechanism by which endocrine response is abated in ESR1-positive breast cancers. Conversely, we delineate that ESR1-responsive enhancer hypomethylation is critical in transition from normal mammary epithelial cells to endocrine-responsive ESR1-positive cancer. Cumulatively, these novel insights highlight the potential of ESR1-responsive enhancer methylation to both predict ESR1-positive disease and stratify ESR1-positive breast cancer patients as responders to endocrine therapy.

## Abstract (web summary)

The molecular factors influencing patient response to endocrine therapy are poorly understood. Here Stone *et al.* characterize the DNA methylome of endocrine response and show that methylation of oestrogen receptor-associated enhancers underpins endocrine sensitivity in human breast cancer.

## Graphical abstract

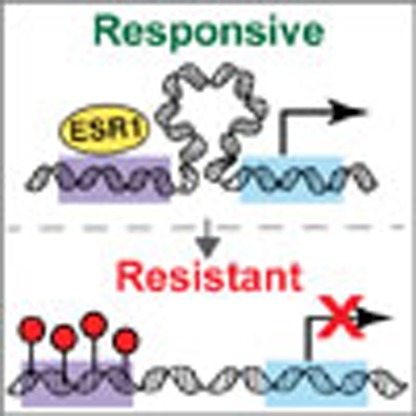

The steroid hormone oestrogen activates the oestrogen receptor (ESR1) to mediate a variety of functions that are central to the normal development and maintenance of multiple tissues^1^. The unique transcriptional response to oestrogen in each tissue-specific cell subtype is, in part, regulated by the epigenome^2^. Differential DNA methylation and chromatin remodelling serve to dictate accessibility to functional, oestrogen-responsive regions of the genome, and thus define endocrine response^3^,^4^. Inappropriate activation of the ESR1 signalling network in mammary epithelial cells initiates neoplastic transformation and drives ESR1-positive breast cancer^1^. Patients with this disease commonly receive adjuvant endocrine therapy, which serves to inhibit ESR1 signalling^1^,^5^. Although endocrine therapy reduces the risk of disease recurrence, a third of patients acquire drug resistance and experience disease relapse^6^. Thus, endocrine sensitivity of both normal breast cells and breast cancer cells is dynamic, raising the hypothesis that global epigenetic reprogramming of oestrogen-responsive regions of the genome can modulate endocrine sensitivity and contributes to the onset of ESR1-positive breast cancer and the acquisition of endocrine resistance.

While recent studies have provided excellent proof of principle that the DNA methylation profile of mammary epithelial cells is altered in early carcinogenesis^7^, and further modified in cell models of endocrine-resistant breast cancer^8^,^9^, they do not address how these changes could directly affect endocrine sensitivity. Here we identify DNA methylation as a key determinant of endocrine response in breast cancer. We show that differential DNA hypermethylation occurs predominantly at oestrogen-responsive enhancer, not promoter regions, and is associated with reduced ESR1 binding and decreased gene expression of key regulators of ESR1 activity. In addition, we demonstrate that the methylation status of these regulatory regions is associated with endocrine resistance in human disease, thus providing a novel mechanism by which endocrine response is abated in ESR1-positive breast cancers.

## Results

### Methylation of enhancer loci in endocrine-resistant cells

**Figure 1:**
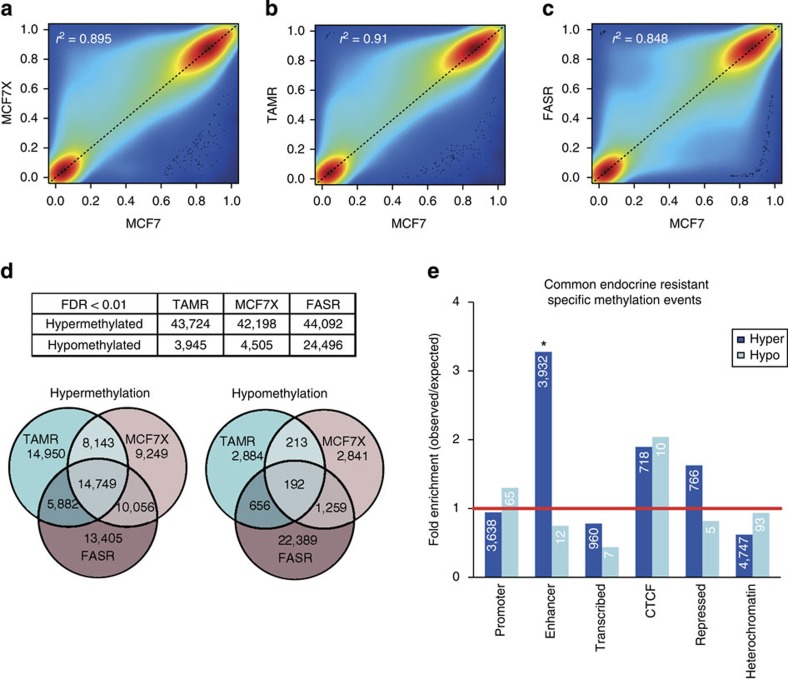
Genome-wide DNA methylation profiling of endocrine-resistant MCF7 cell models. (**a–c**) A colorimetric density plot showing correlation between the HM450 methylation profile of the endocrine-resistant MCF7X (**a**), TAMR (**b**) and FASR (**c**) cells and the parent (endocrine-sensitive) MCF7 cells. The plots show that while the methylation profile of the endocrine-resistant cell lines is strongly correlated with the parent MCF7 cells (MCF7X, *r*^2^=0.895; TAMR, *r*^2^=0.91; FASR, *r*^2^=0.848; Pearson's coefficient), both the MCF7X and TAMR cells predominantly gain DNA methylation, whereas the FASR cells exhibit both hyper- and hypomethylation events relative to parent MCF7 cells. (**d**) A Venn diagram showing the overlap of HM450 methylation probes that are more heavily methylated in multiple endocrine-resistant cells compared with the parent MCF7 cells (FDR<0.01). (**e**) A bar plot showing the association of differentially methylated HM450 probes that were common to all endocrine-resistant cell lines (compared with the parent MCF7 cells) across functional/regulatory regions of the genome as determined by MCF7 ChromHMM annotation^13^. The height of the bars represents the level of enrichment measured as a ratio between the frequency of hypermethylated (dark blue) or hypomethylated (light blue) probes overlapping a functional element over the expected frequency if such overlaps were to occur at random in the genome. Statistically significant enrichments (*P* value<<0.0001; hypergeometric test) are marked with an asterisk. The numbers of commonly hyper/hypomethylated probes located within each specific region are presented in the respective column.

To interrogate DNA methylation remodelling as a critical component of acquired endocrine resistance, we performed methylation profiling in duplicate using the Infinium HumanMethylation 450 beadchip, on ESR1-positive hormone sensitive MCF7 cells, and three different well-characterized endocrine-resistant MCF7-derived cell lines; tamoxifen-resistant (TAMR)^10^, fulvestrant-resistant (FASR)^11^ and oestrogen deprivation-resistant (MCF7X)^12^ cells. Density plots showing the correlation between the DNA methylation profile of parent MCF7 cells and individual endocrine-resistant cell lines indicate that the MCF7X and TAMR cells, which are both ESR1 positive^10^,^12^, predominantly gained DNA methylation as indicated by the increased density of points above the trend line. In contrast, FASR cells, which are ESR1 negative^11^, exhibited both hyper and hypomethylation events relative to parent MCF7 cells as indicated by a symmetrical density distribution (Fig. 1a–c). We first sought to identify the common differential DNA methylation events present in each of the three uniquely derived endocrine-resistant cell models by carrying out paired analyses (that is, each endocrine-resistant cell line versus MCF7 parent control) and overlapping the data (Fig. 1d). We found that across the individual resistant cell lines, 14,749 CpG probes were commonly hypermethylated (false discovery rate, FDR<0.01), whereas only 192 probes exhibited shared hypomethylation (FDR<0.01; Fig. 1d).

**Figure 2:**
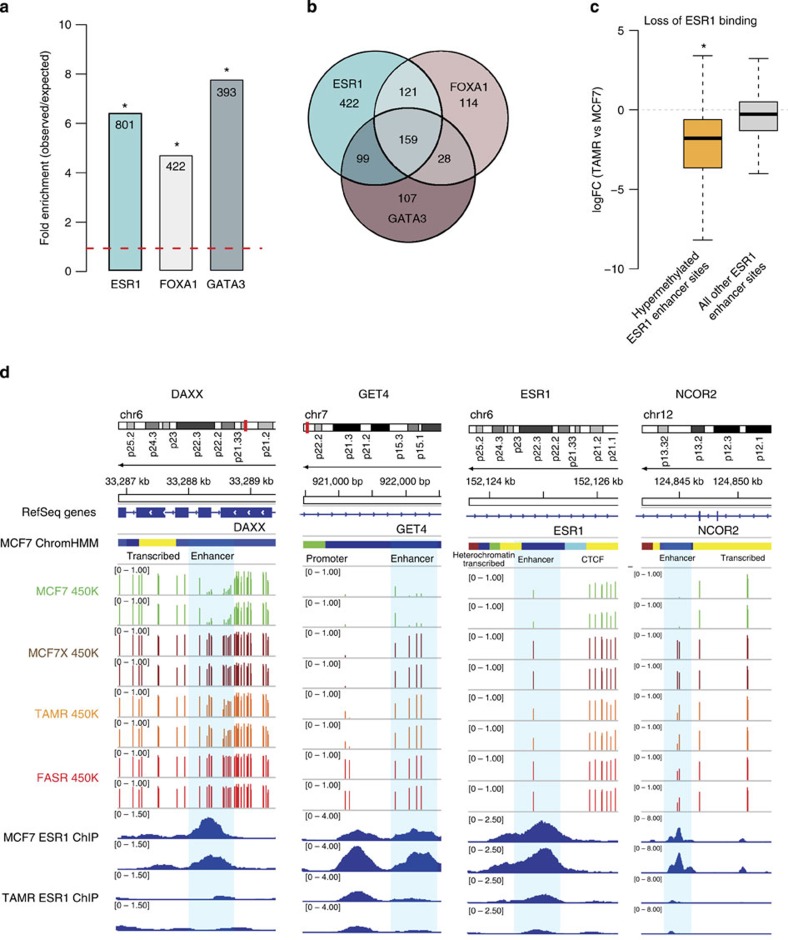
ESR1 regulation of enhancer sites commonly hypermethylated in endocrine-resistant cell models. (**a**) A bar plot showing the association of HM450 probes that were more heavily methylated in endocrine-resistant cell models (compared with MCF7 cells) and also specifically located in enhancer regions, across ESR1-, FOXA1- and GATA3-binding sites in MCF7 cells. The height of the bars represents the enrichment measured as a ratio between the frequency of hypermethylated probes in enhancers overlapping a transcription factor binding site over the expected frequency if such overlaps were to occur at random across the genome (*P value<<0.0001; hypergeometric test). The numbers of commonly hyper/hypomethylated probes located within each specific region are presented in the columns. (**b**) A Venn diagram showing the overlap of enhancer-specific HM450 methylation probes that are more heavily methylated in multiple endocrine-resistant cell models (compared with MCF7 cells) across ESR1-, FOXA1- and GATA3-binding sites. (**c**) A box plot showing the log-fold change (logFC) in ESR1 binding signal at ESR1-enhancer sites that contain at least one commonly hypermethylated probe (yellow box) and all other ESR1-enhancer sites that overlap a HM450 probe (grey box) in TAMR cells compared with the parent MCF7 cells. The mean logFC in ESR1 binding at hypermethylated ER-enhancer sites is −2.29 and the mean logFC of all other ESR1-enhancer sites is −0.52 (*P<<0.0001; *t*-test). (The whiskers of the box plot extend to the most extreme data point, which is no more than 1.5 × interquartile range from the box). (**d**) IGV screen shots to illustrate the loss of ESR1 binding in TAMR cells compared with the parent MCF7 cells in enhancer regions that overlap methylation probes that are more heavily methylated in the endocrine-resistant cell models. The MCF7 ChromHMM regions are colour coded as follows—blue, enhancer; yellow, transcribed; green, promoter; light blue, CTCF; and burgundy, transcribed. The HM450 *β* values are shown for the MCF7 (green), MCF7X (burgundy), TAMR (orange) and FASR cells (red) and are representative of biological duplicates. ESR1 ChIP data (blue) is presented in duplicate for both MCF7 and TAMR cells. The ESR1 enhancers that overlap the regions of endocrine-resistant-specific hypermethylation are highlighted by the blue boxes.

To comprehensively characterize the functional genomic location of differential methylation observed in the endocrine-resistant cell models, we used ChromHMM segmentation of the MCF7 genome (previously described in Taberlay *et al.*^13^; Fig. 1e). Strikingly, significant enrichment of commonly hypermethylated probes was exclusively observed in enhancer regions of the genome (*n*=3,932 probes, *P*<<0.0001; hypergeometric test; Fig. 1e). We next sought to determine whether the enhancer regions identified as being more heavily methylated in all endocrine resistance models were regulated by the ESR1 in the parental MCF7 cells. Using reprocessed, publically available MCF7 ESR1 (ref. 14), GATA3 (ref. 15) and FOXA1 ChIP-Seq data^16^ (two transcription factors closely associated with ESR1 activity), we found that enhancer-specific CpG-hypermethylated probes were enriched in ESR1-binding sites by approximately sixfold, FOXA1-binding sites by fivefold and GATA3-binding sites by eightfold (*P*<<0.0001; hypergeometric test; Fig. 2a). The greatest number of hypermethylated enhancer probes were found to overlap ESR1-binding sites (*n*=801), which represents ∼20% of all hypermethylated probes in enhancer regions. Significantly, 47% (379 out of 801) of the hypermethylated enhancer probes that were located within an ESR1-binding site were also located within a FOXA1 and/or GATA3-binding site (Fig. 2b), which is particularly noteworthy since these transcription factors cooperatively modulate ESR1-transcriptional networks by forming a functional enhanceosome^17^.

### Enhancer DNA hypermethylation and diminished ESR1 binding

Having defined a subset of ESR1-binding sites that overlap enhancer regions which contain hypermethylated loci in multiple models of endocrine resistance (see Methods section; *n*=856 sites, Supplementary Data 1), we sought to determine whether DNA methylation affected the intensity of ESR1 binding at these sites. Using MCF7 and TAMR ESR1 ChIP data^14^, we compared the change in ESR1 binding signal intensity at ESR1-enhancer sites that contained (a) hypermethylated probe(s) to that of all other ESR1-enhancer sites (Fig. 2c). At methylated ESR1-enhancer sites, there was a 2.29-log-fold reduction in ESR1 binding in TAMR compared with MCF7 cells. In contrast, at all other ESR1-enhancer-binding sites, there was a 0.52-log-fold reduction in ESR1 binding in TAMR compared with MCF7 cells. Thus, increased methylation at ESR1-enhancer sites is associated with reduction in ESR1 binding (*P*<<0.0001; *t*-test; Fig. 2c). Four illustrative examples show the loss of ESR1 binding in the TAMR cells at enhancer regions that are more heavily methylated in the endocrine-resistant versus the parent MCF7 (Fig. 2d). The examples include enhancer regions located within the gene body of death-associated protein 6 (*DAXX)*, golgi to ER traffic protein 4 homologue (*GET4*; a member of the BAG6-UBL4A-GET4 DNA damage response/cell death complex^18^), *ESR1* itself and nuclear receptor co-repressor 2 (*NCOR2*; Fig. 2d).

### Enhancer DNA hypermethylation and related gene expression

Since the vast majority of ESR1-enhancer-binding sites identified as hypermethylated in the endocrine-resistant cell lines compared with the parent MCF7 cells were intragenic (that is, 617 out of 856, 72% with at least partial overlap; Supplementary Data 1), we next sought to determine if the DNA methylation of these regions correlated with the expression of the genes in which they were located (or closest TSS if intergenic) in human breast cancer. Using RNA-seq and HM450 methylation data derived from TCGA breast cohort^19^ (*n*=459 patients), we determined that out of the 856 ESR1-enhancer-binding sites of interest, hypermethylation of 328 sites (that is, 38% of ESR1-enhancer sites) correlated with the reduced expression of the genes with which they were most closely associated (Spearman's correlation coefficient; *P*<0.001; Supplementary Data 2). The 328 ESR1-enhancer-binding sites represented 291 unique genes (including those presented in Fig. 2d; Supplementary Data 3). Gene set enrichment analysis revealed that these genes were over-represented in gene sets upregulated by ESR1 activation, downregulated in the acquisition of endocrine resistance and gene sets lowly expressed in basal versus luminal disease, thus suggesting that such genes were critical drivers of oestrogen-driven tumours (Supplementary Fig. 1a). Interestingly, using unsupervised clustering analysis, this gene set (*n*=291) stratifies ESR1-positive and ESR1-negative breast cancer patients (Supplementary Fig. 1b). Cumulatively, this indicates that the methylation events occurring throughout the acquisition of endocrine resistance are serving to facilitate an oestrogen-independent phenotype reflective of a breast cancer subtype that is refractory to endocrine therapy.

### ESR1-enhancer methylation defines breast cancer subtype

**Figure 3:**
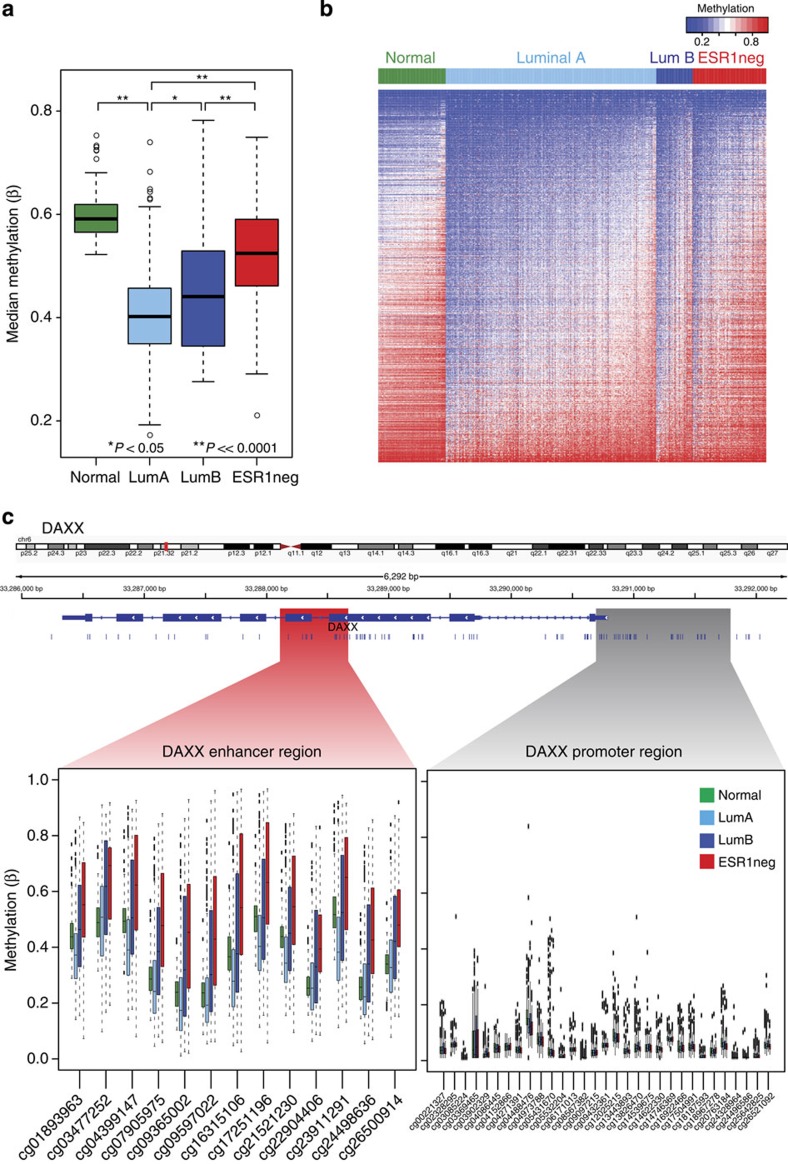
Association between ESR1-enhancer methylation and breast cancer subtype. (**a**) A box plot showing the median methylation of all HM450 probes that overlap an enhancer region, an ESR1-binding site and demonstrate hypermethylation in endocrine-resistant versus parental MCF7 cells (*n*=801 probes), in normal breast tissue (green; *n*=97), luminal A (light blue; *n*=301), luminal B (dark blue; *n*=52) and ESR1-negative (red; *n*=105) breast cancer (data obtained from TCGA breast cancer cohort; *P<0.05, **P<<0.0001; Mann–Whitney *U-*test). (The whiskers of the box plot extend to the most extreme data point, which is no more than 1.5 × interquartile range from the box). (**b**) A heatmap showing the methylation profile of 801 ESR1-enhancer-specific HM450 probes that are more heavily methylated in endocrine-resistant versus parent MCF7 cells in normal breast tissue (green; *n*=97), luminal A (light blue; *n*=301), luminal B (dark blue; *n*=52) and ESR1-negative (red; *n*=105) breast cancer. Columns are patient samples and rows are HM450 probes. The level of methylation is represented by a colour scale—blue for low levels and red for high levels of methylation. (**c**) Box plots showing distribution of methylation *β* values in normal *n*=97 (green), luminal A (light blue; *n*=301), luminal B (dark blue; *n*=52) and ESR1-negative (red; *n*=105) breast cancer samples across HM450 probes overlapping the ESR1-binding site located within the DAXX enhancer (Chr6: 33288112-33288670; left panel) and the DAXX promoter region (1,000 bp upstream and 100 bp downstream of the transcription start site; Chr6: 33290693-33291793; right panel). (The whiskers of the box plots extend to the most extreme data point, which is no more than 1.5 × interquartile range from the box).

We next sought to determine whether ESR1-enhancer hypermethylation was indicative of breast cancer subtype. We assessed the median methylation of all hypermethylated ESR1-enhancer probes (*n*=801) in TCGA normal (*n*=97), luminal A (*n*=301), luminal B (*n*=52) and ESR1-negative (*n*=105) patient HM450 data (Fig. 3a). In normal breast tissue (which is reported to be ∼7% ESR1 positive^20^), the median methylation of the ESR1-enhancer sites was highest, while median DNA methylation was significantly reduced in luminal A disease (*P*<<0.0001; Mann–Whitney *U-*test), which is indicative of its endocrine-responsive state. Interestingly, median ESR1-enhancer methylation was greater in luminal B patients compared with luminal A patients (*P*=0.017; Mann–Whitney *U-*test), who are almost twice as likely to acquire endocrine resistance^21^. In ESR1-negative disease, median methylation was higher than in luminal disease (versus luminal A, *P*<<0.0001; versus luminal B, *P*<<0.0001; Mann–Whitney *U*-test; Fig. 3a). A heatmap highlights the hypomethylated status of the ESR1-enhancer sites in luminal A disease relative to normal breast tissue and the other breast cancer subtypes (Fig. 3b). This trend is clearly illustrated at the *DAXX* enhancer region in which each CpG within the ESR1-binding site was hypomethylated in luminal A disease compared with normal tissue and luminal B and ESR1-negative cancer (Fig. 3c). Critically, no such variability was apparent at the *DAXX* promoter region (1,000 bp upstream and 100 bp downstream of the transcription start site; Fig. 3c), suggesting a significant regulatory effect of increased methylation at the enhancer locus.

### ESR1-enhancer hypermethylation predicts endocrine failure

**Figure 4:**
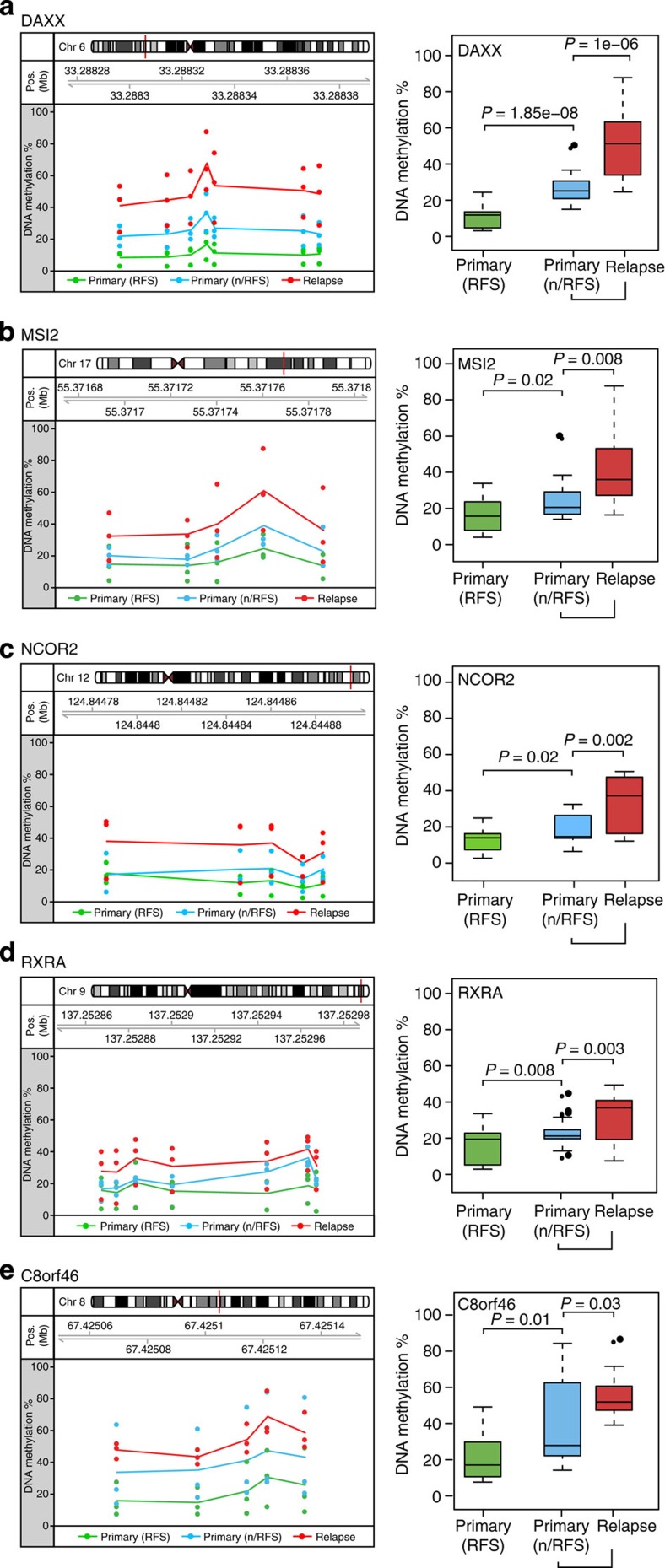
ESR1-enhancer DNA hypermethylation in acquired endocrine resistance in human breast cancer. (**a–e**) (Left panel) A scatter plot showing the methylation of individual CpG sites across the ESR1-enhancer region of interest ((**a**)-*DAXX*—Chr6: 33288296-33288372; (**b**)-*MSI2*—Chr17: 55371693-55371786; (**c**)-*NCOR2*—Chr12: 124844786-124844883; (**d**)-*RXRA*—Chr9: 137252867-137252967; (**e**)-C8orf46—Chr8: 67425069-67425134) in three primary luminal A breast cancers from patients that received adjuvant endocrine therapy and exhibited RFS (green), three primary luminal A breast cancers from patients that relapsed following adjuvant endocrine therapy, defined as no n/RFS (blue) and their matched local relapse (red). Each dot represents the % methylation at an individual CpG site for a single patient and the lines represent the average methylation for the region in primary RFS (green), primary n/RFS (blue) and matched recurrent tumours (red). (Right panel) Box plots showing the distribution of methylation values across the ESR1-enhancer region depicted in the left panel for RFS (green), prognosis/RFS (blue) and matched recurrent tumours (red); *P* values correspond to *t*-test comparison between RFS versus n/RFS, and n/RFS versus relapse tumours. (The whiskers of the box plots extend to the most extreme data point, which is no more than 1.5 × interquartile range from the box).

Given that ESR1-enhancer hypermethylation is prevalent in acquired endocrine resistance *in vitro* (Figs 1e and 2a-d) and in molecular subclassifications of breast cancer that are intrinsically less responsive to endocrine therapy (Fig. 3a–c), we next sought to determine the methylation status of a panel of these loci in ESR1-positive (luminal A) breast cancer samples from patients with different outcomes. Primary samples were sourced from patients who received endocrine therapy for 5 years and either experienced relapse-free survival (RFS; >14 years) or those who had relapsed (<6 years), defined as no relapse-free survival (n/RFS). Matched local relapse samples were also compared with the primary n/RFS patient samples. All patients received the same endocrine therapy (tamoxifen; anonymized patient data is given in Supplementary Data 4). Using a multiplex bisulphite-PCR resequencing methodology specifically devised for formalin-fixed, paraffin-embedded (FFPE)-derived DNA^22^, the methylation of multiple CpG sites across a panel of nine oestrogen-responsive enhancer regions was interrogated (technical duplicate correlates for all amplicons investigated are shown in Supplementary Fig. 2). These enhancer regions included those located within *DAXX*, *MSI2*, *NCOR2*, *RXRA* and *C8orf46* (Fig. 4a–e) and enhancer regions located within *GATA3*, *ITPK1*, *ESR1* and *GET4* (Supplementary Fig. 3a–d). The assay was repeated with DNA extracted from biological duplicates of the endocrine-resistant cell lines and the parent MCF7 cells to ensure its viability (Supplementary Fig. 4a–i; technical duplicate correlates for all amplicons investigated are shown in Supplementary Fig. 5). The average methylation levels detected at all enhancer loci were significantly higher in the recurrent tumours compared with the matched primary (n/RFS) tumours (*DAXX*, *P*<0.0001; *ESR1*, *P*<0.0005; *RXRA*, *P*<0.005; *GET4*, *NCOR2*, *GATA3*, *MSI2*, *P*<0.01; *C8orf46*, *ITPK1*, *P*<0.05; *t*-test), confirming that DNA methylation at ESR1-responsive enhancers is acquired in resistant disease (Fig. 4 and Supplementary Fig. 3). The difference in DNA methylation between RFS and n/RFS primary tumours was less considerable, although a statistically significant difference was observed for *DAXX*, *P*<0.0001; *RXRA*, *P*<0.01; *C8orf46*, *P*=0.01; *NCOR2* and *MSI2* (*P*<0.05; *t*-test) enhancer regions (Fig. 4).

## Discussion

Our results support a model whereby ESR1-responsive enhancer DNA methylation is a fundamental unifying characteristic that defines endocrine sensitivity in breast cancer. Interestingly, previous studies interrogating DNA methylation changes in endocrine-resistant cell models have predominantly reported ESR1-regulated promoter methylation^8^,^9^,^23^,^24^,^25^,^26^. Our study is the first to combine in depth MCF7 ChromHMM annotation and genome-wide methylation data from multiple resistance models to more comprehensively characterize global differential methylation across diverse genomic regions. We show for the first time that the methylation status of enhancers is associated with the inhibition of ESR1 binding *in vitro* and with the reduced expression of critical regulators and effectors of ESR1 activity in human disease. The identification of ESR1-responsive enhanceosome hypermethylation is both novel and considerably pertinent in the context of endocrine resistance, since genome-wide positional analyses defining the set of *cis*-regulatory elements that recruit ESR1 in breast cancer cells have revealed its predominant recruitment to enhancers as opposed to promoter regions^3^,^27^,^28^,^29^,^30^. Enhancers are more common than promoters in the mammalian genome^31^ and can regulate gene transcription from tens to thousands of kilobases away by promoting communication with target promoters through chromatin looping^32^,^33^. In our study, the majority of ESR1-regulated enhancer regions identified as hypermethylated in the resistant cells were located within gene bodies. Strikingly, hypermethylation of these enhancer regions was frequently correlated with reduced expression of the host gene, which is in line with recent studies that have shown that over half of all enhancer regions are located within a gene body and that the activation of these enhancers can indeed affect the transcription of the host gene^34^,^35^. Examples of genes whose expression inversely correlated with ESR1-enhancer DNA methylation include *DAXX* and *GET4*, which have been previously associated with roles in apoptosis^18^,^36^. It is conceivable that the loss of expression of genes associated with pro-apoptotic functions facilitates the progression of endocrine resistance by reducing the efficacy of apoptotic signalling pathways activated by endocrine therapies^37^.

Importantly, the ESR1-responsive enhancer hypermethylation events identified in the endocrine-resistant cell lines were also differentially methylated in endocrine-sensitive and endocrine-resistant breast cancer patient samples. Therefore, it is feasible that ESR1-responsive enhancer methylation status is reflective of endocrine dependence and could potentially be used to stratify patients as responders to endocrine therapy. For example, *NCOR2*, a gene whose expression has previously been associated with metastasis-free survival in 620 lymph node-negative patients with ESR1-positive breast cancer^38^, was shown to negatively correlate with ESR1-enhancer methylation. In the present study, *NCOR2* enhancer methylation was significantly higher in the poor (non-relapse-free) prognosis patients, compared with the good (relapse-free) prognosis primary luminal A breast cancer patients. Critically, however, in matched recurrent tumours, enhancer DNA methylation was further increased, supporting the hypothesis that the endocrine-resistant methylation profile is acquired, rather than pre-existing, limiting its potential prognostic value. Intriguingly, it could be a combination of both acquired and intrinsic methylation differences that give rise to endocrine-resistant disease. One possible explanation is that sparse, or ‘seeding' methylation at ESR1-responsisive enhancer sites in primary tumours could reflect a propensity to gain extensive methylation that spreads as resistance develops, which then becomes firmly established in recurrent disease (as discussed in ref. 39). Further characterization of ESR1-responsive enhancer methylation in endocrine-resistant disease will hereafter be an important area of future investigation, as will be the assessment of its potential predictive and prognostic application in breast cancer.

## Methods

### Cell culture and HumanMethylation450K array

MCF7 breast cancer cells and the corresponding endocrine-resistant sub-cell lines were kindly given to our laboratory by Dr Julia Gee (Cardiff University, UK). In brief, MCF7 cells were maintained in RPMI-1640-based medium containing 5% (v/v) fetal calf serum (FCS). TAMR MCF7 cells were generated by the long-term culture of MCF7 cells in phenol red-free RPMI medium containing 5% charcoal stripped FCS and 4-OH-tamoxifen (1 × 10^−7^ M; TAM). FASR MCF7 cells were generated by the long-term culture of MCF7 cells in phenol red-free RPMI medium containing 5% charcoal stripped FCS and fulvestrant (1 × 10^−7^ M; FAS). Long-term oestrogen-deprived MCF7 (MCF7X) cells were generated by the long-term culture of MCF7 cells in phenol red-free RPMI medium containing 5% charcoal stripped FCS. Endocrine-resistant sub-lines were established and characterized following 6 months endocrine challenge/oestrogen deprivation exposure^10^,^11^,^12^. All cell lines were authenticated by short-tandem repeat profiling (Cell Bank, Australia) and cultured for <6 months after authentication. Genomic DNA was extracted using the Qiagen DNeasy Blood and Tissue kit according to the manufacturer's instructions. HumanMethylation450K arrays were carried out by the Australian Genome Research Facility (AGRF; Melbourne, Australia).

### HM450 analysis

Two biological replicates per condition—MCF7, TAMR, MCF7X or FASR—were profiled on Illumina's HumanMethylation450K array. Raw HM450 data was preprocessed and background normalized with the Biconductor minfi package^40^ using preprocess Illumina(..., bg.correct=TRUE, normalize='controls', reference=1); resulting *M* values were used for statistical analyses and *β* values for heatmap visualizations and clustering. Differential methylation analysis of the preprocessed data was performed using the Bioconductor limma package.

### Genomic segmentation and annotation

The ChromHMM segmentation of the MCF7 genome was obtained from Taberlay *et al.*^13^. Enhancer (‘Enhancer' and ‘Enhancer+CTCF') and Promoter categories (‘Promoter', ‘Promoter+CTCF' and ‘Poised Promoter') were collapsed into a single ‘Enhancer' and ‘Promoter' state respectively for the purposes of our analysis. RefSeq transcript annotations were obtained from UCSC genome browser^41^,^42^.

### ChIP-seq data acquisition and analysis

ESR1 ChiP-seq data for ESR1 in MCF7 and TAMR^14^ was utilized in this study. Reads were mapped to genome build HG19 (GRCh37) with bowtie and mismatched (>3 mismatched bases), multiple mapping and duplicate reads were excluded from downstream analysis. ESR1 enrichment peaks were identified with the HOMER software suite^43^ using the findPeaks utility (-style factor -fragLength 200 -size 300 -F 0 -L 0 -C 0 -poisson 1e-06) on each experiment separately. We merged the resulting peaks to produce a ground set of 120,735 regions for subsequent analysis. Active ESR1 regions were identified in MCF7 by comparing the distribution of reads overlapping the ground set of ESR1 regions in the three MCF7 ESR1 experiments (GSM798423, GSM798424 and GSM798425) and MCF7 input experiment (GSM798440) with edegR^44^. This yielded 54,265 active ESR1 regions in MCF7 (FDR<0.05). A similar strategy was applied to TAMR data to yield 49,511 ESR1 regions in TAMR cells. Regions of differential ESR1 binding were identified by comparing the distribution of sequence reads in MCF7 and TAMR across the ground set of ESR1 regions using edgeR and potential variation in copy number was accounted for using DiffBind^14^. This analysis resulted in 24,711 regions with statistical significant gain (FDR 5%) and 32,343 regions with statistical significant loss (FDR 5%) of ESR1 binding in TAMR cells as compared to MCF7 cells. ESR1 peaks overlapping HM450 probes were assigned to the nearest RefSeq transcript (<20 kb distance) for the purposes of gene expression analysis. Raw MCF7 GATA3 and FOXA1 ChIP-Seq data was obtained from Theodorou *et al.*^15^ and Hurtado *et al.*^16^, respectively. Data were processed in the same manner as outlined for ESR1 ChIP-seq above.

### Gene set enrichment analysis

GSEA was performed against the Molecular Signatures Database v4.0 (MSigDB)^45^ C2 Collection. Enrichment was assessed by hypergeometric testing as implemented in the R stats package.

### TCGA data acquisition

DNA methylation analysis utilized clinical data available through the TCGA Breast Invasive Carcinoma cohort^19^. Raw HM450 methylation data (level 1) were obtained from the TCGA data portal (normal samples=97, ESR1-positive tumours=353 and ESR1-negative tumours=105). ESR1-positive tumours were further divided into luminal A (lumA=301) and luminal B (lumB=52) populations using progesterone receptor (PR) expression, such that lumA were ESR1+/PR+ and lumB were ESR1+/PR−. Processed RNA-Seq expression data (level 3) were obtained from TCGA data portal (588 ESR1 positive tumours with 73 matched normals and 174 ESR1 negative samples with 19 matched normals).

### Multiplex bisulfite-PCR resequencing of clinical FFPE DNA

Bisulfite DNA conversions were performed using a manual protocol. For each conversion, ∼100 ng was bisulfite converted at a time. Conversion took place at 80 °C for 45 min in the presence of 0.3 M NaOH, 3.75 mM quinone and 2.32 M sodium metabisulfite, as per the method of Clark *et al.*^46^. The multiplex bisulfite-PCR reaction was performed as follows^22^. In brief, Promega HotStart GoTaq with Flexi buffer (M5005) was used with the following components at the indicated concentrations: 5 × green (1 × ), CES 5 × , (0.5 × , N.B. refer to ref. 47 for CES recipe), MgCl_2_ (4.5 mM), dNTP's (200 μM each), primers (forward and reverse at 100 mM), Hot Start Taq (0.025 U μl^−1^), DNA (2 ng μl^−1^ ). All primers used are listed in Supplementary Data 5. Cycling conditions were: 94 °C, 5 min; 12 cycles of (95 °C, 20 s; 60, 1 min); 12 cycles of (94 °C, 20 s; 65 °C, 1 min 30 s); 65 °C, 3 min, 10 hold. Agencourt XP beads were using to clean-up and concentrate the multiplex reaction for subsequent barcoding (that is, addition of Illumina p5/p7 sequences and sample-specific DNA barcodes). The barcoding PCR used the following reagents at the indicated final concentrations in a 100-μl reaction: 1 × GoTaq Green Flexi buffer; 0.25 × CES; 4.5 mM MgCl_2_; 200 μM dNTPs; 0.05 U μl^−1^ HotStart Taq; 25 μl of pooled template after Agencourt XP bead clean-up; and 20 μl MiSeq (Fluidigm PN FLD-100-3771). Cycling conditions were: 94 °C, 5 min; 9 cycles of (97 °C, 15 s; 60 °C, 30 s; 72 °C, 2 min); 72 °C, 2 min; 6 °C, 5 min. MiSeq sequencing was performed used the MiSeq Reagent Kit v2, 300 cycle; PN MS-102-2002. Bioinformatic analysis started with adaptor trimming using Trim galore (options: --length 100). Mapping used the Bismark methylation mapping programme^48^ running Bowtie2 (ref. 49) (options: --bowtie2 -N 1 -L 15 --bam -p 2 --score L,-0.6,-0.6 --non_directional; bismark_methylation_extractor -s -merge_non_CpG –comprehensive --cytosine_report). To reduce computational overhead, mapping took place against only those genomic regions which were being investigated, plus an additional 100 bp–1 kb of flanking sequence.

### Clinical sample acquisition and DNA extraction

FFPE breast cancer samples were obtained from the St George Hospital, Kogarah, Australia (Ethics approval reference from St George Hospital Human Research Ethics Committee is 96/84). The deidentified haematoxylin–eosin-stained sections were reviewed by a pathologist and representative tumour areas were marked and blocks were cored accordingly. Genomic DNA was extracted using the Qiagen AllPrep DNA/RNA FFPE kit according to the manufacturer's instructions.

## Additional information

**Accession codes:** Cell line HumanMethylation450K array data is available online at GEO (GSE69118).

**How to cite this article:** Stone, A. *et al.* DNA methylation of oestrogen-regulated enhancers defines endocrine sensitivity in breast cancer. *Nat. Commun.* 6:7758 doi: 10.1038/ncomms8758 (2015).

## Supplementary Material

Supplementary Figures Supplementary Figures 1-5

Supplementary Data 1 ESR1 binding sites that contain hypermethylated loci in multiple models of endocrine resistant breast cancer.

Supplementary Data 2 Correlation of DNA hypermethylation at ESR1 enhancer loci and differentail gene expression in TCGA breast cohort.

Supplementary Data 3 Genes whose expression negatively correlate with increased ESR1 enhancer DNA hypermethylation in multiple models of endocrine resistant breast cancer (n = 291 unique genes).

Supplementary Data 4 Clinical parameters of patient samples used.

Supplementary Data 5 Primer Sequences.
