# Supplementary material for: DNA methylation of oestrogen-regulated enhancers defines endocrine sensitivity in breast cancer
Source: Nat Commun. 2015 Jul 14;6:7758. doi: 10.1038/ncomms8758 (PMC4510968; doi:10.1038/ncomms8758)
Supplement: Supplementary Figures — 1-5 [file ncomms8758-s1.pdf]

Supplementary Figure 1

a.

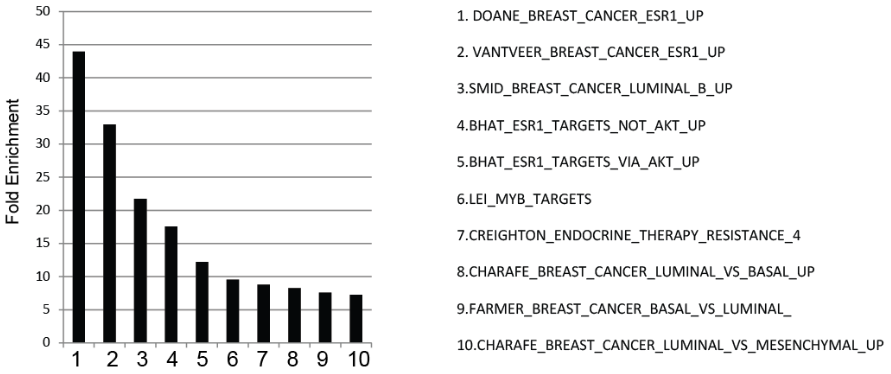

b.

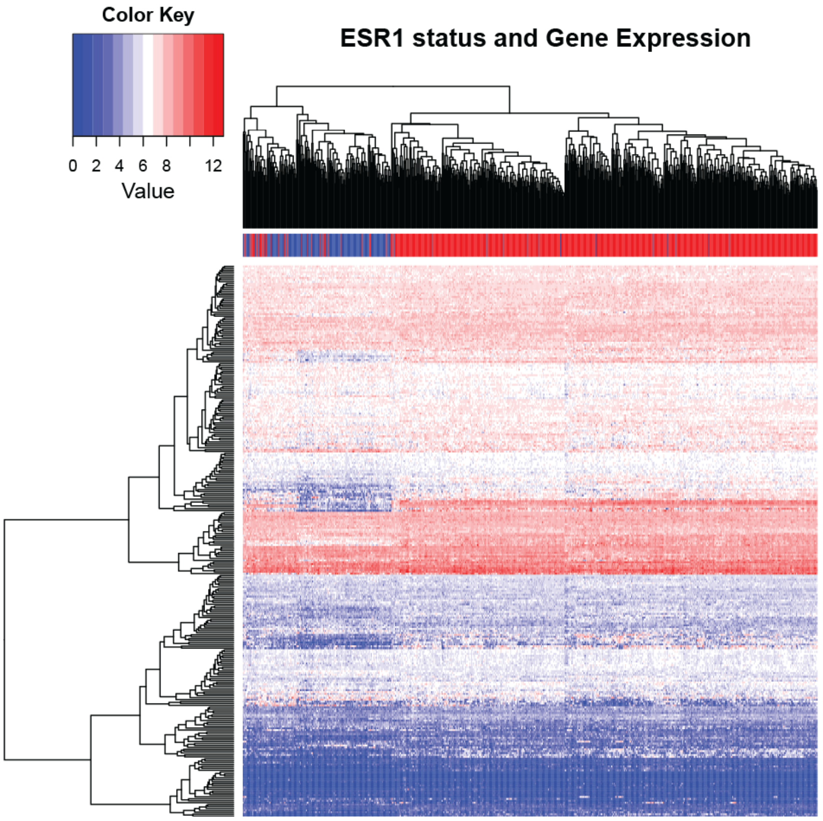

Supplementary Figure 1. Characterisation of genes whose expression is negatively affected by ESR1-enhancer hypermethylation in human breast cancer. (a) Hypergeometric testing of genes whose expression is negatively affected by ESR1-enhancer hypermethylation in human breast cancer (n = 291) in the MSigDB C2 database. The height of the bars represents the level of enrichment measured as a ratio between the number of genes overlapping an MSigDB C2 gene set over the expected frequency if such overlaps were to occur at random in the genome (p-value << 0.0001; hyper-geometric test). (b) Unsupervised clustering of the gene set whose expression is negatively correlated with ESR1-enhancer methylation (n = 291) in ESR1 positive (red) (n = 174) and ESR1 negative (n = 588) breast cancer patients (obtained from TGCA breast cohort RNA-seq data).

Supplementary Figure 2

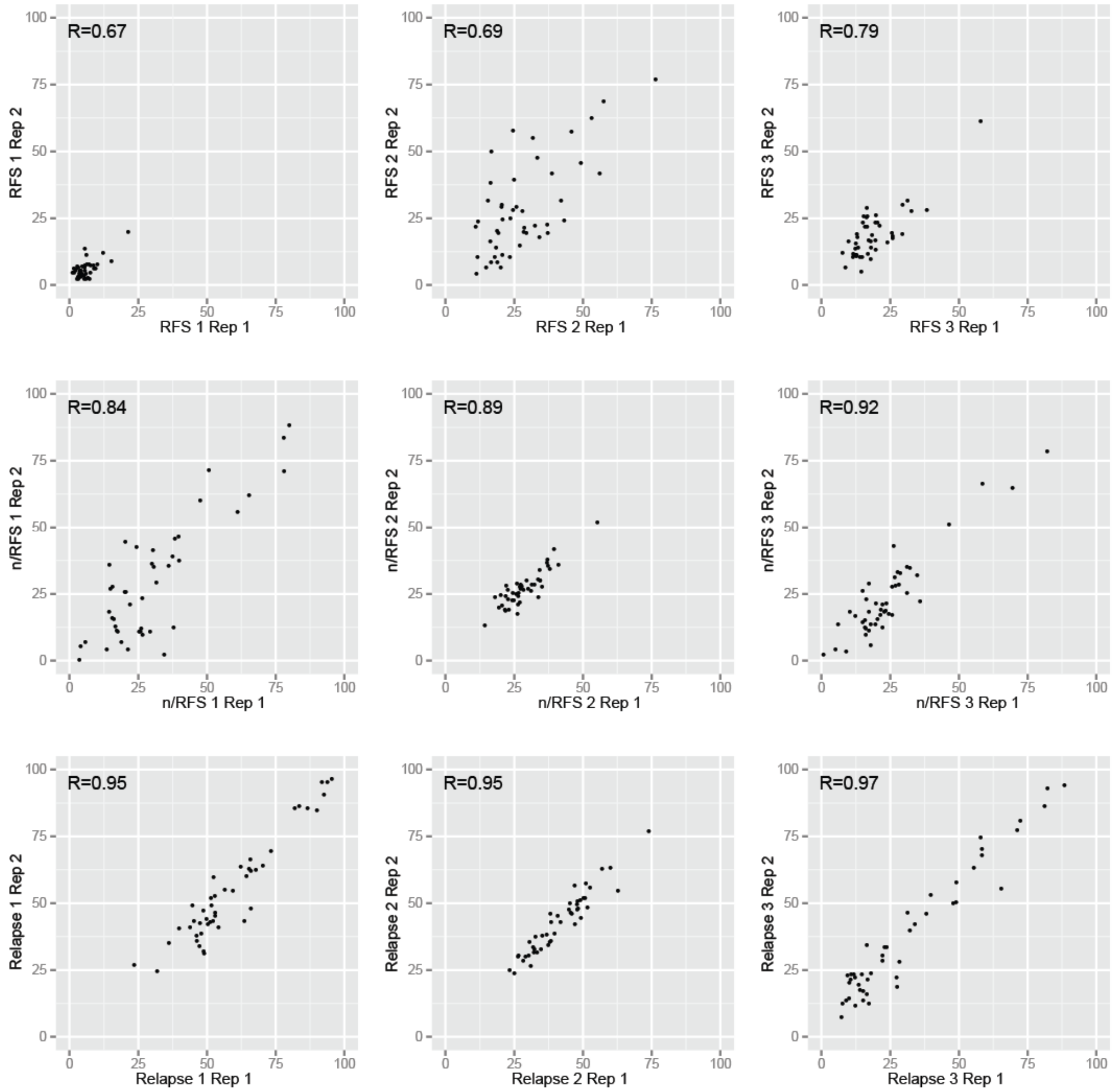

Supplementary Figure 2. Graphical representation of the correlation between the technical replicates presented in Figure 4. Scatter plots showing the correlation between the technical replicates of multiplex bisulphite-PCR resequencing data presented in **Figure 4** (R = Pearson correlation).

Supplementary Figure 3

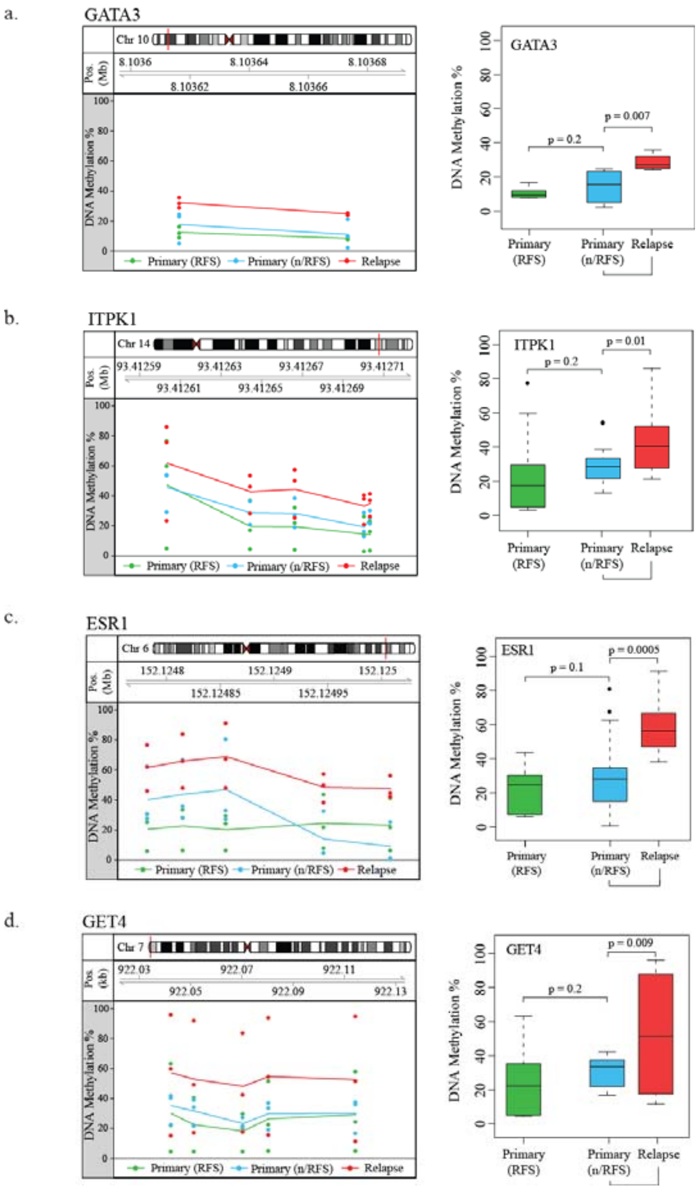

Supplementary Figure 3. ESR1-Enhancer DNA hypermethylation in acquired endocrine resistance in human breast cancer. (a-d) (Left panel) A scatter plot showing the methylation of individual CpG sites across the ESR1-enhancer region of interest (**a-** *GATA3* – Chr10: 8103616-8103673; **b-** *ITPK1* - Chr14: 93412603-93412703; **c-** *ESR1 - Chr6: 152124782-152125008*; **d-** *GET4* - Chr7: 922042-922114) in 3 primary luminal A breast cancers from patients that received adjuvant endocrine therapy and experienced relapse free survival (RFS) (green), 3 primary luminal A breast cancers from patients that relapsed following adjuvant endocrine therapy (n/RFS) (blue) and their matched local relapse (red). Each dot represents the % methylation at an individual CpG site for a single patient and the lines represent the average methylation for the region in primary RFS (green), primary n/RFS (blue) and matched recurrent tumours (red). **(Right Panel)** Box plots showing the distribution of methylation values across the ESR1-enhancer region depicted in the left panel for RFS (green), prognosis/RFS (blue) and matched recurrent tumours (red); p-values correspond to t-test comparison between RFS vs n/RFS, and n/RFS vs relapse tumours. (The whiskers of the boxplot extend to the most extreme data point, which is no more than 1.5xIQR from the box).

Supplementary Figure 4

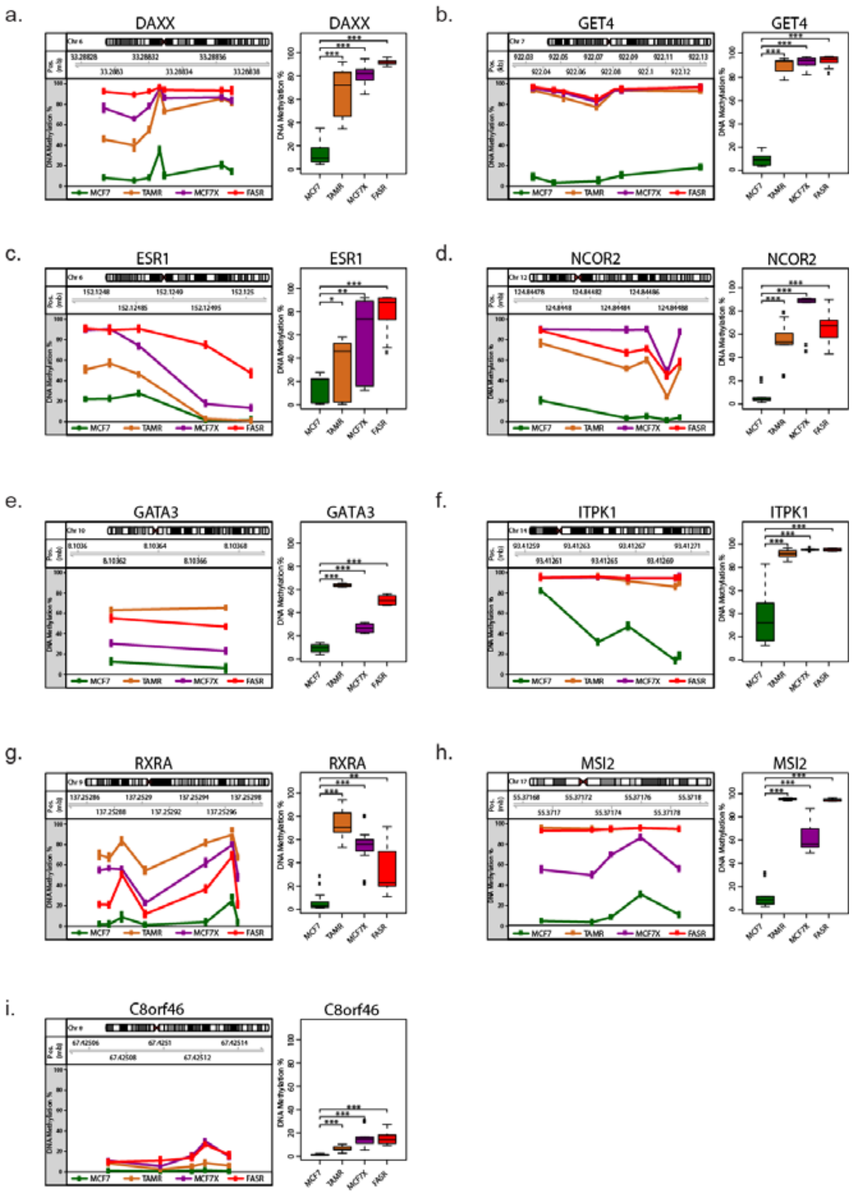

Supplementary Figure 4. ESR1-Enhancer DNA hypermethylation in cell models of acquired endocrine resistance. (a-I) (Left panel) A scatter plot showing the methylation of individual CpG sites across the ESR1-enhancer region of interest (a- *DAXX* - Chr6: 33288296-33288372; **b-** *GET4* - Chr7: 922042-922114; **c-** *ESR1* - Chr6: 152124782-152125008; **d-** *NCOR2* - Chr12: 124844786-124844883; **e-** *GATA3* – Chr10: 8103616-8103673; **f-** *ITPK1* - Chr14: 93412603-93412703; **g-** *RXRA* - Chr9: 137252867-137252967; **h-** *MSI2* - Chr17: 55371693-55371786; **i-** *C8orf46* - Chr8: 67425069-67425134) in the parental MCF7 cells (green), and the endocrine resistant derivatives, TAMR (orange), MCF7X (purple) and FASR (red). Each dot represents the % methylation at an individual CpG site and the lines represent the average methylation for the region. **(Right Panel)** Box plots showing the distribution of methylation values across the ESR1-enhancer region depicted in the left panel for the parental MCF7 cells (green), and the endocrine resistant derivatives, TAMR (orange), MCF7X (purple) and FASR (red) (mean ± SD) (*p < 0.05, **p < 0.01, ***p < 0.001; t-test). (The whiskers of the boxplot extend to the most extreme data point, which is no more than 1.5xIQR from the box.)

Supplementary Figure 5

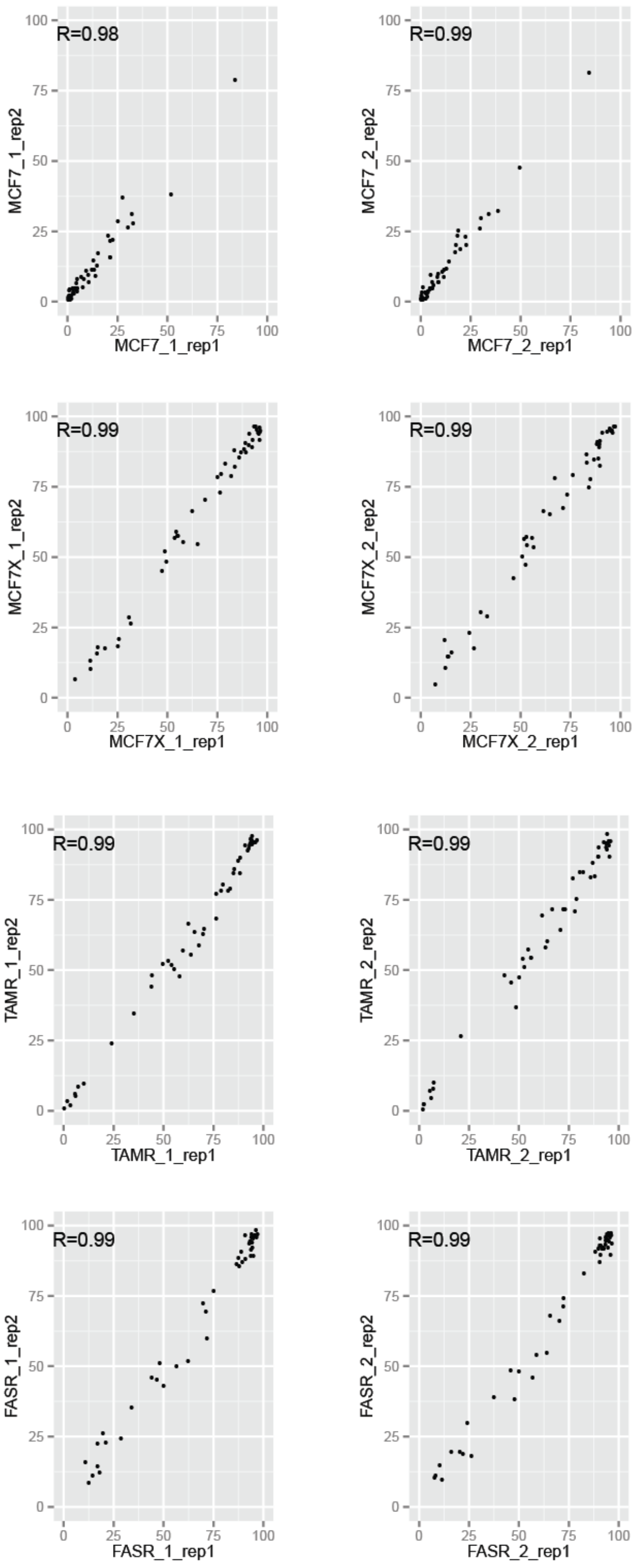

Supplementary Figure 5. Graphical representation of the correlation between the technical replicates presented in Supplementary Figure 4. Scatter plots showing the correlation between the technical replicates of multiplex bisulphite-PCR resequencing data presented in **Supplementary Figure 4** (R = Pearson correlation).
